# Evaluation of regional project to strengthen national health research systems in four countries in West Africa: lessons learned

**DOI:** 10.1186/s12961-017-0214-8

**Published:** 2017-07-12

**Authors:** Issiaka Sombié, Jude Aidam, Gabriela Montorzi

**Affiliations:** 1 West Africa Health Organisation, BP 153, Bobo-Dioulasso, Burkina Faso; 2 Council on Health Research for Development (COHRED), Geneva, Switzerland

**Keywords:** Guinea-Bissau, Liberia, Sierra Leone, Mali, National health research system, Post-conflict, Health systems strengthening, West Africa

## Abstract

**Background:**

Since the Commission on Health Research for Development (COHRED) published its flagship report, more attention has been focused on strengthening national health research systems (NHRS). This paper evaluates the contribution of a regional project that used a participatory approach to strengthen NHRS in four post-conflict West African countries – Guinea-Bissau, Liberia, Sierra Leone and Mali.

**Methods:**

The data from the situation analysis conducted at the start of the project was compared to data from the project’s final evaluation, using a hybrid conceptual framework built around four key areas identified through the analysis of existing frameworks. The four areas are governance and management, capacities, funding, and dissemination/use of research findings.

**Results:**

The project helped improve the countries’ governance and management mechanisms without strengthening the entire NHRS. In the four countries, at least one policy, plan or research agenda was developed. One country put in place a national health research ethics committee, while all four countries could adopt a research information management system. The participatory approach and support from the West African Health Organisation and COHRED were all determining factors.

**Conclusion:**

The lessons learned from this project show that the fragile context of these countries requires long-term engagement and that support from a regional institution is needed to address existing challenges and successfully strengthen the entire NHRS.

## Background

In 1990, the report from the Commission on Health Research for Development (COHRED) stated that “*strengthening research capacity in developing countries is one of the most powerful, cost-effective, and sustainable means of advancing health and development*” [1]. Since then, more attention has been paid to national health research systems (NHRS) in developing countries [2–18], and several initiatives to strengthen these systems have taken place in Africa [10, 13–15]. A national research system is an important element of a modern health system that defines the environment in which research is encouraged and carried out. WHO defines it as a system that provides governance, capacity building, knowledge generation and mechanisms to use evidence, supported by sustainable funding mechanisms for a country’s health systems to drive research activities [6]. Finally, it brings together all research stakeholders, from researchers, research managers and donors, all the way to potential research users (decision-makers, civil society, professional associations).

Within the Economic Community of West African States (ECOWAS), the West African Health Organisation (WAHO) is the specialised institution responsible for health. Its mission is to help the countries of this community offer their populations the best quality of care. To accomplish this, it must promote health research to ensure that decisions are evidence based. Strengthening national research systems in the region is a key step in this promotion of research. In 2009, with the support of technical partners, WAHO organised a regional workshop to analyse the national research systems of the 15 ECOWAS countries [19]. The results indicated that these systems had some weaknesses, marked by the absence of research policies, plans and agendas; the absence of a framework or mechanism for coordinating stakeholders; the absence or poor functioning of national ethics committees; the absence of monitoring and evaluation mechanisms; poor individual and institutional capacities for managing and carrying out research; poor collaboration among researchers; poor dissemination; and poor use of research findings in decision-making. The analysis also showed that three countries (Guinea-Bissau, Liberia and Sierra Leone) were far behind compared to the others. What they had in common was that they were all post-conflict countries with few qualified human resources, a significant number of competing priorities, many stakeholders to help with reconstruction [20–25], more interest in offers of aid than in research, an insecure environment, and limited research funding [26–28]. To address these needs, a regional project focusing on strengthening national research systems was developed in these three countries. Mali was also added because, although it has many research institutions, it suffered from poor coordination among stakeholders [29].

This project, which ran from 2011 to 2015, was implemented by WAHO with technical support from COHRED. The project was implemented in four stages, composed of a situation analysis to better understand the national research systems of the various countries; identification of priorities and activities to be put in place in the countries; implementation of the chosen activities; and finally, the project evaluation. The project adopted a participatory process that included stakeholders from the beneficiary countries, as well as other existing partners in the field. It began with a launch workshop to which three representatives from each country were invited. These representatives included the person in charge of research in the Ministry of Health and in the Ministry of Education, Science and Technology, and a director of a public health research institution. During this meeting, NHRSs in the four countries were described using data from the 2009 meeting, and complementary information was gathered. The teams from each country undertook a mapping exercise on the various health research stakeholders to identify the existing potential. The teams also identified priorities for improving the NHRS in each country. Finally, the HRWeb [30, 31] platform developed by COHRED was presented as a research information management tool. The stakeholder mapping and priority identification exercises and the implementation activities were validated during the project information workshop in each of the four countries in 2011, and the teams received technical and financial support.

This paper attempts to evaluate whether the activities of this regional project strengthened the NHRS in these four countries. We use a hybrid conceptual framework built around four key areas identified through the analysis of existing frameworks [2, 16–18] and the priorities identified during the workshop in 2009. Given that this project was developed by WAHO, the lessons learned were also used to make suggestions regarding the role of a regional institution and other technical and financial partners in strengthening NHRS.

## Methods

The study is based on a comparative evaluation before and after the intervention. The ‘before intervention’ information comes from the situation analysis conducted at the start of the project to better describe the national intervention systems. The end-of-project data is taken from the final project evaluation report, while the project information comes from the project protocol and implementation reports.

### Analysis of the national research system situation in the four countries

The situation analysis at the start of the project was carried out using three information sources. The first was a review of the literature associated with interviews with participants from the four countries in the 2011 regional project launch workshop [27]. The other sources were two studies carried out in the various countries during workshops to validate the plans developed during the launch workshop. They were used to analyse research information management and capacity-building training in the four countries [32, 33].

### External project evaluation

To analyse the situation at the end of the intervention, we used the report from the external evaluation [34] conducted by an independent expert at the end of the project, who performed document review and qualitative analysis. The document review consisted of an analysis of the project protocol, activity implementation plans, and technical and financial activity and project implementation reports. This document review analysed project planning, intervention strategies, planned activities and their implementation, resources used, results obtained, and observations made to identify strengths, weaknesses, opportunities and threats or challenges that were noted during the project implementation.

The qualitative study consisted of 50 direct interviews through Skype or by telephone, using an interview guide. The individuals interviewed were the WAHO management team, WAHO heads of departments, services and programmes, people in charge of health research for the Ministry of Health, heads of research institutions in the countries, focal points and HRWeb-country developers, WAHO-country focal points, people responsible for networks of researchers in the countries, and contacts in WAHO’s partner international institutions for the implementation of the project, namely the International Development Research Centre and COHRED. The interviews addressed what did and did not work in the implementation, what was and was not done well, what needed to be improved in the future and the lessons learned.

Triangulation of all the data from the document review and a qualitative analysis identified the project’s major findings, indirect findings, challenges and lessons learned, to review the role of WAHO and other stakeholders and improve the national research systems in post-conflict countries.

### Conceptual project analysis framework

To strengthen NHRS’s capacities, several conceptual frameworks have been proposed. The research capacity conceptual framework created by McIntyre [16] describes the dimensions through which health research capacities can be strengthened nationally. These are individual and organisational dimensions that include government (research platforms and technologies), key government departments, universities, university hospitals, non-governmental organisations (NGOs), agencies of the United Nations system, research management structures, public health institutes, private industry, and regulatory structures (pharmaceutical quality control committee and ethics committees). The conceptual framework in Pang et al. [2], or the WHO framework, was built on the four essential roles that an NHRS should play, namely stewardship, financing, resource creation and support, and production and dissemination of research results. Finally, the COHRED conceptual framework was developed to help countries build a strong system with the capacities needed to produce optimal research results [17]. The COHRED conceptual framework has four components, namely foundation (policy development, definition of priorities and management), resources (capacities, funding and coordination), optimisation (ethical regulation, civil society commitment, fair research contracting, pharmaceutical innovation, research communication, research information system and evaluation follow-up), and favourable political environment (leadership) (Table 1).

**Table 1:** Comparative analysis of the different conceptual frameworks for national health research system strengthening

Domain	Pang et al. [2] framework	McIntyre [16] framework	COHRED [17] framework
Governance and management	Stewardship (define and articulate a vision for national health research system; identify appropriate health research priorities and coordinate adherence to them; set and monitor ethical standards for health research and research partnership; monitor and evaluate health research system)	National research environment (governance and coordination, ethical guidelines/standards) Task network (Ministry of Heath, international funders, Southern NGO, Northern universities)	Foundations (development of policies, definition of priorities and management) Resources (coordination) Optimisation (ethic regulation, civil society engagement, research information system, and monitoring and evaluation) Enabling political environment (leadership)
Capacities	Creating and sustaining resources (build, strengthen and sustain the human and institutional capacities to conduct, absorb and utilise health research)	Individual (training and experiences, disciplines, research, management and leadership skills) Institutional (critical mass, external environment/infrastructure, collegial environment, leadership, funding)	Resources (capacities)
Financing	Financing (secure research funds and allocate them accountably)	National research environment (funding demand for research outputs) External research environment (international funders, international organisations)	Resources (financing) Optimisation (fair research contracting)
Diffusion and use of research results	Producing and using resources (produce scientifically valid research; translate and communicate research to inform policy, strategies, practices and public opinions; promote the use of research to develop a new tools, e.g. drugs, vaccines, devices, to improve health)		Optimisation (research communication)

Analysis of these frameworks shows the need to work in four fields (governance and management, capacities, funding, and dissemination/use of the research results) to strengthen a national research system. For this project, we built a framework from these four fields. Indicators have been put forward by others to assess the progress in relation to each of them [2, 18].

Governance is the government’s duty to observe and to build a coalition, an accountability system, and a system for regulating all health research that takes place in the private and public sectors [18]. Therefore, it assumes that there are policies, plans, priorities, legislation, regulatory texts and regulatory mechanisms such as ethics committees. Management allows for the implementation of national guidelines based on a regulatory framework and for the purpose of tracking processes. This will require a research information management system that collects data for building predefined indicators. Therefore, the indicators in the governance and management field were policy availability and implementation, strategic plans, research agendas, the existence of a national health ethics committee, a research coordination mechanism, and a research information management system.

Capacity strengthening is defined as a process of transferring skills and capacities to individuals, institutions, organisations or a nation that will allow them to define and prioritise problems systematically, to develop and assess solutions scientifically, and to share and apply the knowledge generated [16, 35]. For Lansang et al. [35], additional short- and long-term individual, institutional and country approaches may be applied. These include basic or postgraduate training, learning by doing, promotion of partnerships among institutions from developed and developing countries, and creation of centres of excellence. Therefore, the indicators selected in this field include the existence of individual capacity-strengthening programmes (research methodology, ethics, research management, research use and dissemination) to manage, guide and share research results, the number of people who benefited from these capacity strengthening programmes, and the number of institutional capacity strengthening activities.

The funding function refers to estimating financial needs, mobilisation, and making available and managing funds from the government and internal and external funders at the individual, institutional and organisational levels to make the NHRS work [18]. Availability of a line in the budget to support a health ministry’s research acknowledges the importance of research and a government’s commitment to support it. Therefore, in this project, in the wake of the various international commitments, such as devoting 2% of budgets to health research, the indicator used was availability of a line for research in the health ministry’s budget.

The research use and dissemination function includes disseminating the research results and applying them to defending, planning, developing and implementing policies and plans and to following up on evaluations. To achieve success in this dissemination and application, knowledge platforms are required and they must convey, summarise and communicate the research results to provide information on policies and practices [18]. These platforms can be knowledge transfer platforms, a discussion forum for researchers and decision-makers, and a national publication base. Here, the existence of frameworks for discussions between researchers and decision-makers, in which they can share and discuss research results and their use, and which will create a space for dialogue between these actors, has been chosen as an indicator.

## Results

### Research governance and management

During the situational analysis phase, the four countries that signed the Algiers Declaration on Research for Health [36] and the Libreville Declaration on Health and Environment [37] had health policies or plans that gave high priority to research development, showing a willingness to develop health research [27]. This willingness was more notable in Guinea-Bissau, which had requested support from COHRED in 2008 [28]. The health research governance structure was based in the health ministries in three countries, while in Mali, it was in the ministry of higher education and scientific research. In three countries, these structures were at the management level, but in Liberia, they were at the unit level [11]. In general, these structures were led by people with no training in research management and they had no more than three officers. Only Mali had a policy document and a research development plan. None of the countries had a mechanism for coordinating the various research stakeholders. Liberia was the only country with no national ethics committee, but had two institutional ethics committees, one of which acted as a national ethics committee; nevertheless, there was no effective coordination between them. All the countries had documents that could be used to regulate research. None of the four countries had a centralised research information management mechanism or health research stakeholder mapping.

The priorities selected varied between countries. The Liberia team prioritised the promotion of a culture of use of research findings among policy and decision-makers, the mobilisation of funding for health research, setting up a national ethics committee, establishing a national agenda for governance, and the development of a research information management system to be supported through HRWeb. Sierra Leone’s priorities were aimed at developing a health research policy and plan, strengthening ethics review and human resources for research for health, and mobilising financial resources for research for health. The Guinea-Bissau team presented a work plan setting goals and activities, starting with a research for health system mapping, and setting the basis for good governance, priority setting, ethics committee building, as well as advocacy for sustainable funds. It also proposed a capacity-building process, including financial management and accountability, communication, networking and monitoring the use of research results. Just before the inception workshop, Mali adopted a National Health Research Policy and Plan of Action, a long-term vision to improve the health research system and ultimately reduce disease incidence and mortality. The team proposed a work plan linked to the above document, including the creation of a health research coordination committee, improvement of the ethical committees, fund mobilisation strengthening, and ultimately achieving a better dissemination and use of research findings, training of human resources, infrastructure building or renovating, the development of computer-based systems and networking, as well as advocacy to dedicate 2% of the national health budget and 5% of donor funds for health programmes to research for health.

In Liberia, the project has given the impulse for the creation of a Health Research Unit within the Ministry of Health and Social Welfare that will manage and coordinate research for health in the country. Liberia’s draft system map, plan of work and research agenda were validated during a workshop held in December 2011. Suggestions were made regarding the roles of individuals and institutions, and thus the roles of some institutions were expanded. A roadmap was drafted for the development of a national research programme for health policy that will provide the country with a policy framework and guidelines for the conduct of research.

In Sierra Leone, the work plan was reviewed and validated by local interested parties at a meeting convened by the Ministry of Health in 2011. A consultant supported by the ChRAIC Irish African Partnership for Health Research Capacity Strengthening project, coordinated the development and completion of the national research programme for health policy. With support from that project, an inter-sectoral group developed and reviewed both documents. The group was composed of the same member constituencies represented on the governing body. The Guinea-Bissau’s draft system map, plan of work and research agenda were validated during a workshop held in November 2011. The project supported the finalisation of a research agenda and its translation into English. In Mali, during a stakeholders meeting, the action plan and health research system map were validated with minor changes and the terms of reference of the national coordination committee was developed and submitted to the Health Minister for the creation of the committee.

Table 2 shows the changes in research governance and management mechanisms in the four countries by the end of the project. Each country has maps of the various health research stakeholders, providing information about who these are and what roles they play. We can see that the various health research stakeholders come from ministries of health, education, science and technology, environment, and agriculture and livestock, as well as from NGOs, the United Nations system, universities, funders and major research initiatives. These stakeholders in each country were categorised as information producers, research regulators, and research consumers. A policy, a strategic plan and two research agendas or priorities were then developed. In Guinea-Bissau and Liberia, research priorities had been finalised, adopted and shared with all stakeholders. However, neither the policy nor the plan developed in Sierra Leone to strengthen the national research system were adopted. Development of a health policy in Liberia began with technical and financial contributions from the WHO national office, but the process was interrupted with the outbreak of the Ebola virus epidemic. Recently, the health ministry expressed the need for support to finalise this document. In Mali, unfortunately, the research dialogue committee’s terms of reference had not been ratified nor had the committee been put in place. A national health research ethics committee was implemented in Liberia and consisted of people who had received ethics training. Regarding research information management, each of the four countries had a page on the HRWeb platform and a regional page had also been created. A link was created between the sites of the health ministries and WAHO and the HRWeb platform. In each of the four countries and in WAHO, people received permission to enter information on the platform on behalf of the countries and of WAHO. A list of 24 indicators was then developed and generated directly by the platform on the WAHO page, allowing countries to be compared in terms of governance, regulatory institutions, research institutions, civil society organisations and research projects [38].

**Table 2:** Countries NHRS situation before and after the regional project implementation according to the framework domains

Domains	Indicators	Guinea-Bissau		Liberia		Mali		Sierra Leone	
		Before	After	Before	After	Before	After	Before	After
Governance and management	Existence of a map of the research for health stakeholders	No	Yes	No	Yes	No	Yes	No	Yes
	Existence of research policy, plan and agenda	No	Yes	No	Yes	Yes	Yes	No	Yes
	Existence of research coordination system	No	No	No	No	No	No	No	No
	Existence of national ethical committee	Yes	Yes	No	Yes	Yes	Yes	Yes	Yes
	Existence of health research monitoring system and indicators	No	Yes	No	Yes	No	Yes	No	Yes
Capacities	Existence of training programme in health research	Yes	Yes	Yes	Yes	Yes	Yes	Yes	Yes
	Number of institutional capacities strengthening activities				1				
	Number of persons trained in ethical review		1		26		1		
	Number of persons trained in research management MoH		1		2				
	Number of persons trained in research methodology		18		32		2		8
	Number of persons trained to Master level in research		0		1		1		0
	Number of persons trained to use HRWeb platform		20		24		28		28
Financing	Existence of budget lines within the MoH to finance research	No	No	No	No	Yes	Yes	No	No
Diffusion and use research results	Existence of research results dissemination mechanisms	No	No	Yes	Yes	No	No	Yes	Yes

*MoH* Ministry of Health

### Capabilities

Upon analysis of the situation, there was, in general, limited availability of human resources for health in post-conflict countries. This limited availability was even more marked in the field of research, where the number of people with PhDs was low, except in Mali. Regarding ethics committees in the four countries, not all members had received training. The situational analysis showed that all countries offered training in research methodology that varied greatly in terms of duration, content and instructors. At the institutional level, it was noted that not all the bodies in charge of research had the capabilities needed to function. All the countries had expressed the need for trainings in the fields of ethics, research management and research methodology, as well as on how to use the HRWeb information management platform and on how to increase institutional capabilities.

Considering the various training needs, the project reviewed existing short-term trainings in research methodology and in ethics. This review revealed that a great deal of non-standardised training exists, as do challenges in keeping training going year after year. The project itself used three training approaches. The first consisted of short-term training sessions that only required officers to be away from their workstation for a short time. The second was support for training through university degrees. Bursaries were offered by WAHO through a call for candidacies and were open to nationals from all ECOWAS countries, or by request by a specific country. The third training approach was to promote an online research management training programme that was taking place in Latin America in Spanish. Short-term trainings were organised in research methodology, in ethics and in how to use the HRWeb platform. The research methodology instructors were regional and national individuals with research experience, whereas the instructors on ethics and on how to use the HRWeb platform were experts from the COHRED group. The trainings on research methodology and on use of the HRWeb platform were opened to all other ECOWAS countries.

On an institutional level, the WAHO and COHRED teams helped those in charge of research management better understand their role through discussions and through sharing documents about NHRS management. In Liberia, these teams also helped develop terms of reference for the research unit.

At the end of the project, the short-term trainings had allowed a total of 245 people to receive training (39 in Guinea-Bissau, 29 in Mali, 81 in Liberia and 37 in Sierra Leone), in research ethics, in managing research programmes, in research methodology and in use of the HRWeb platform (Table 2). The people trained were stakeholders in charge of research management at the Ministry of Health and at research institutions, as well as researchers, heads of health programmes and computer scientists. Three people (two in Mali and one in Liberia) obtained their Master’s degrees in epidemiology (Table 2). The person trained in Liberia was a research management officer, and the two people trained in Mali worked in a hospital and at the Ministry of Health. Two participants had enrolled in the online training, but due to Internet connectivity issues, they had to abandon it without completing their training.

One direct impact of these trainings is the implementation of Liberia’s national research ethics committee, whose members include those individuals who took the ethics training organised as part of the project. Another is the existence of information available via the HRWeb platform. In September 2014, 279 research documents were available on the platform (including policy documents, legislation, plans and research agendas for the various countries), along with 118 research projects reviewed by ethics committees, 226 research institutions (including five in Guinea-Bissau, 24 in Mali, eight in Liberia and five in Sierra Leone) and a list of approximately 40 managers authorised to enter information, which is a testament to the platform’s use. One indirect result of the project was the appropriation and use of the platform in Benin and Niger, which continues even today. After the research methodology training, a weak point was noted regarding beneficiaries in post-conflict countries who, upon returning, had often not completed their research protocol, as compared to other participants from other countries in the area.

By contrast, it should be noted that the training promoted by the project did not meet all the countries’ needs. Additionally, only three people could benefit from diploma courses, which is insufficient when compared to needs. Furthermore, the choice of who to train was not under the project’s control. Finally, training alone does not provide all the capabilities necessary for performance. Material and equipment needs, as well as the need to create an institutional environment, were not under the project’s control.

### Funding

At the start of the project, Mali was the only country with a dedicated research budget at the Ministry of Health. However, even in Mali, it was still difficult for researchers to access funds due to considerable administrative red tape.

The main priority for each country was to improve access to funding at the Ministry of Health, and the strategy adopted during the project to achieve this goal was advocacy. During their visits to the four countries, teams from WAHO and COHRED advocated with authorities from the Ministry of Health every time they met. Research service heads also advocated with their immediate supervisors.

At the end of the project, no improvement was noted in this regard, and Mali remained the only country to have a dedicated research budget. However, in Liberia and Sierra Leone, complementary funding was accessible through WHO and through an NGO, respectively, to help cover the fees for the consultants who had helped develop the policy and the research plan.

### Dissemination and use of research

At the start of the project, only Sierra Leone had a framework for researchers and decision-makers to exchange ideas and which supported knowledge transfer and the use of research findings. A group of researchers worked with the Ministry of Health to organise a biomedical research symposium, where research findings were presented and discussed and research training was provided. In Liberia, time was allotted during the monthly Health Sector Coordination Committee meeting to discuss research findings, and there was a directory of research carried out in the country. The strategy employed by the project was to motivate country stakeholders to create these frameworks for dialogue or support existing frameworks. In Sierra Leone, the project supported 2 years of the biomedical research symposium. At these two fora, protocol writing and article writing workshops were organised for researchers. In Liberia, the Research Unit created a database of research carried out in the country. No framework for discussion between researchers and decision-makers was created.

The project outcomes show differences among countries, likely due to the local contexts and the leadership, will and commitment of those implementing the project in the different countries. Table 2 shows that the highest number of results were obtained in Liberia, where the context was more favourable and those implementing the project were committed. The project began around the same time as the creation of the Research Unit, which consisted of motivated, results-oriented young professionals, and therefore the project was an opportunity for them. Moreover, WHO’s office in the country was committed to research development. It provided the Research Unit with material support, professionals participated in national meetings, and a consultant was supported to develop policy documents and research plans. In addition, there was no turnover in the Research Unit, making it possible to implement almost all planned activities. On the other hand, in Sierra Leone and Guinea-Bissau, over the 4 years of the project, the bodies in charge of research in the Ministry of Health, which was responsible for implementing the project, went through two and three different heads, respectively. In addition, in 2012, there were coups in Mali and Guinea-Bissau, resulting in the suspension of travel and funding in Mali. The events in all three of these countries hindered the implementation of activities. Liberia, despite support from WHO and the project, was unable to finish developing and validating its research policy and plan. Development of this document began in 2013, and despite a variety of technical and financial support, the issue of technical capacity was a limiting factor both with the national consultant and in the Research Unit. Technical capacity also played a determining role in Sierra Leone and Guinea-Bissau. In Sierra Leone, two people oversaw research and the implementation of the project. In Guinea-Bissau, the departure of the first head, who was skilled in research management, affected the implementation of the project. Some of the people trained to run HRWeb needed motivation before they would continue entering information into the online platform. The low rates of Internet access and connectivity in the post-conflict countries (Guinea-Bissau, Liberia and Sierra Leone) influenced the use of HRWeb to manage research information. In conclusion, for context to be favourable, it is necessary to have leadership, commitment, individual and institutional capacities, financial resources and stakeholder commitment.

The successes achieved are also partly due to the project and the presence of the WAHO and COHRED teams, whose contribution was recognised and well received by stakeholders in the countries, as demonstrated by this statement: “*The WAHO team did everything they could. They were always at our side. The ball is in the country’s court to change things*.” The country teams were pleased with this support, as shown in this quotation from the final project report: “*The research teams from all four countries studied expressed unanimous satisfaction with the commitment and capacities of the members of the WAHO Research Unit in their support for the countries implementing or strengthening their HR systems. Their availability and professionalism were especially appreciated. Observation of the dynamics and operation of the team during the WAHO investigation further confirmed this assessment*” [32].

## Discussion

The project demonstrated that a systematic, participatory approach to developing national research governance and management mechanisms can work, even in post-conflict countries. At the same time, capacity building for an entire national research system is a significant task requiring long-term commitment. The project helped improve the national environment in four countries, but did not address all the local challenges that impact the overall strength of a NHRS. The lessons learned include the fact that WAHO’s supporting role was both necessary and appreciated by the countries.

The weaknesses identified in the four NHRSs can be grouped under the following issues: governance, management, funding, individual and institutional capacities, leadership, collaboration, coordination, and dissemination and use of research findings. These issues were targeted in the conceptual framework and corroborate those identified in other studies in Africa [6, 8, 11, 12, 14].

The choice of priorities for NHRS capacity building differs from country to country. Experiments with NHRS capacity building in other African countries [10, 14, 35] have shown that the needs and priorities in this area can differ from country to country, highlighting the importance of basing any project on local stakeholders’ priorities. This approach also has the benefit of greater involvement of local stakeholders, as we observed when implementing our project. The commitment of stakeholders in the four countries is what allowed us to make a map of research stakeholders, identify their priorities and activities, and implement them.

The project outcomes were the development of governance and management mechanisms like policies, plans and research agendas, the implementation of a national ethics committee, and the adoption of a research information management system (HRWeb). These kinds of successes have been documented in other countries [10, 14, 35], and show that it is possible to make a few qualitative changes in national research systems in Africa, especially in post-conflict countries, where research can assist good decision-making to support planning. Kirigia et al. [14] demonstrated a general trend toward improvement of national research systems in Africa by comparing the results of three studies performed in 2003, 2009 and 2014, respectively. Despite these improvements, post-conflict countries in West Africa were still rated poorly in 2014 [18]. Out of the 15 ECOWAS countries, Mali was ranked fourth, Liberia twelfth, Guinea-Bissau thirteenth, and Sierra Leone fourteenth, highlighting the need for long-term work in these countries.

In the context of this project, the following factors may have contributed to some of the results. The project activities helped create a foundation and improve knowledge of all research stakeholders through mapping, increase knowledge of the state of the NHRSs based on analyses of the situation, raise awareness and motivate different stakeholders through regional and national workshops, motivate stakeholders for change by involving them in priority identification and implementation activities and exposing them to other countries’ experiences, create dialogue and trust among stakeholders through a variety of meetings, and support change through technical and financial support to help the country teams carry out activities and build capacities. These actions contributed to gaining a good understanding of the current state of the NHRS, implementing a body in charge of steering, management and coordination, defining stakeholders’ roles, and implementing a network to bring all stakeholders together, harmonise them, and build their capacities. These were the factors identified as determinant in experiments in South Africa, Kenya, Malawi, Tanzania and Zambia [10, 15, 36]. The countries involved appreciated the presence of the WAHO and COHRED teams, and the partners’ contributions to the countries helped enact change. This shows the importance of supporting country teams, which often have significant weaknesses in terms of their capacities and face numerous challenges. Support helps teams overcome challenges, motivates them to move forward and helps them negotiate with partners. In general, the project helped inform, educate and motivate local stakeholders around the real NHRS situation through analyses of the situation, motivated stakeholders to commit to making changes by having them choose priorities, and helped local stakeholders create change by making technical and financial support available to them and helping them implement activities. These project actions created a favourable environment for policies, plans, research agendas and ethics committees, but did not introduce all the foundations for a broader national research system, such as leadership and sufficient individual and institutional capacities; these require long-term actions. This shows the importance of technical partners in helping stakeholders in countries.

Despite the contributions of the project, overall capacity building for the NHRSs was not achieved. Analysis of the implementation of the project in each country showed that, in addition to the contributions of the project, deficiencies in leadership, project ownership by stakeholders, a supportive environment, individual and institutional capacities, financial capacity, and difficult socio-political environment all played a role, demonstrating the importance of the local context. In most of these countries, the context was one of limited number and quality of human resources, high turnover rates, motivation problems, and bureaucracy. These factors are known to contribute to low performance in most developing countries [39–45], and are more pronounced in post-conflict countries [46–53]. The existence of these factors affects project implementation and suggests that short-term projects cannot strengthen all NHRSs, especially in post-conflict countries. These countries will need long-term support to help them find solutions to these complex problems.

While delegating tasks to personnel with few qualifications has been proposed as an approach to improve healthcare in post-conflict countries, this is not possible in research, which requires qualified personnel. Inter-sectoral collaboration with departments of education, science and technology, academic institutions, research institutions, and NGOs should be considered in post-conflict countries as a partial solution to capacity problems. Another strategy would be to develop a capacity-building programme using the ‘learning by doing’ method, which would have the advantage of keeping workers in the field and reaching numerous people.

While the project was being implemented, experience sharing with other countries was promoted and noted as a determining factor in East, Southern and South Africa [10]. In the long run, such an activity can be carried out by a regional institution such as WAHO. This organisation could also help negotiate with donors to obtain long-term funding and support their implementation. Through this regional project, WAHO demonstrated that it can play a supporting role in post-conflict countries in West Africa. Its role as a facilitator [54] includes leadership promotion, marketing, strategic communication, policy advocacy, coordination, networking, resource mobilisation support, harmonisation support, and partnership development. These forms of support help countries develop and implement capacity-building programmes adapted to their situations. Currently, WAHO is partly playing this role through regional projects funded by the World Bank (WARDS [55], SWEDD [56] and REDISSE [57]) in the fields of disease surveillance and family planning promotion, building WAHO’s supporting role for countries. WAHO’s supporting role in research is seen positively by some post-conflict countries. At the end of the Ebola virus epidemic, the three countries most heavily affected in West Africa decided to collaborate on research and asked for WAHO’s support [58]. WAHO should answer this call to strengthen its role with countries. WAHO should improve its role by adding negotiation and mediation to its functions to help countries obtain long-term capacity-building programmes from donors. It could also use a portion of the funds from these projects to ensure regional collaboration, assessment monitoring, and policy and technical support. There are mechanisms that could help it play its role, such as the ECOWAS forum on best practices in health, the Annual Assembly of Health Ministers, intra-country technical meetings, and national and regional research exchange frameworks. The opportunities for post-Ebola research capacity building in some of these post-conflict countries must be acted upon to involve all stakeholders in developing adapted NHRS capacity-building programmes that can be supported by all stakeholders in these countries.

In addition to WAHO’s role, the involvement of other stakeholders, like United Nations agencies, with WHO as a lead, is crucial. By having a permanent presence and activities in these countries, these institutions are already helping to strengthen national research systems by providing support, for example, in the implementation of the project in Liberia and in funding research activities. They can help with advocacy, funding and technical implementation. The World Bank, the African Development Bank and other research donors should also provide key funding to implement these capacity-building projects for national health research systems.

## Conclusion

This evaluation showed that a participatory regional project can help effect positive change in NHRS governance and management. However, long-term capacity building can only be achieved through long-term engagement.

## Abbreviations

COHRED: Commission on Health Research for Development
ECOWAS: Economic Community of West African States
NGO: non-governmental organisation
NHRS: National health research system
WAHO: West African Health Organisation.
